# Ultra-High-Field MRI Morphometry of the Lateral Geniculate Nucleus in Patients with Advanced Visual Loss Due to Late-Stage Retinitis Pigmentosa

**DOI:** 10.3390/brainsci16040354

**Published:** 2026-03-26

**Authors:** Katarzyna Nowomiejska, Aleksandra Czarnek-Chudzik, Anna Niedziałek, Robert Rejdak, Radosław Pietura, Kamil Jonak

**Affiliations:** 1 Department of General and Pediatric Ophthalmology, Medical University of Lublin, 20-079 Lublin, Poland; aleksandra.czarnek-chudzik@umlub.edu.pl (A.C.-C.); robert.rejdak@umlub.edu.pl (R.R.); 2 Department of Radiography, Medical University of Lublin, 20-093 Lublin, Poland; anna.niedzialek@umlub.edu.pl (A.N.); radoslaw.pietura@umlub.edu.pl (R.P.); 3 Department of Technical Computer Science, Faculty of Mathematics and Information Technology, Lublin University of Technology, Nadbystrzycka 36B, 20-618 Lublin, Poland; k.jonak@pollub.pl

**Keywords:** retinitis pigmentosa, visual pathway, lateral geniculate nucleus

## Abstract

**Highlights:**

**What are the main findings?**

- The volume of the lateral geniculate nucleus (LGN) has decreased in patients with advanced retinitis pigmentosa.
- There is a correlation between the volume of the left LGN and the right eye best-corrected visual acuity in the advanced RP group.

**What are the implications of the main findings?**

- A 7 Tesla MRI scan enables the imaging of the LGN—essential structure of the visual pathway.
- Neuroimaging of the brain may be a useful tool in retinitis pigmentosa patients to assess the efficacy and indication for treatment with future methods.

**Abstract:**

**Background/Objectives**: To investigate the volume of the lateral geniculate nucleus (LGN) in patients with advanced retinitis pigmentosa (RP). **Methods**: Nineteen patients with advanced RP (mean age 52 years and mean duration of illness 357 months) and twenty-one age-matched normal subjects have been examined using 7 Tesla MRI of the brain. Brain segmentation was carried out with the “recon-all” function in the FreeSurfer software (version 7.4.1) package. **Results**: The volumes of the left and right LGN were significantly smaller in those patients with RP, in comparison to the controls. We found a significant positive correlation between the volume of the left LGN and the right eye best-corrected visual acuity in the RP group. **Conclusions**: Late-stage RP leads to a significant reduction in LGN volume, as measured with 7 Tesla MRI. The volume of the left LGN was correlated with the visual function of the right eye in patients with late-stage RP. The statistical power of the results is limited by there being a low number of RP patients included in this study, which is due to the low prevalence of this rare eye disease.

## 1. Introduction

Retinitis pigmentosa (RP) is the most common form of inherited retinal dystrophy; it is characterized by progressive degeneration of rods and cones, and leads to night blindness and, ultimately, complete vision loss [1]. RP is one of the leading causes of bilateral blindness in young adults, with an incidence of 1 in 3000 people worldwide. The disease is caused by mutations in one of over 150 genes that play a part in the phototransduction, cell trafficking and rhodopsin recycling pathways [2]. The genes associated with these diseases produce altered protein products that have downstream effects in pathways critical to vision, including different pathways such as phototransduction, the visual cycle, photoreceptor development, cellular respiration, and retinal homeostasis [3]. The multitude of genotypes is not reflected in the phenotypes, and making the diagnosis is based on characteristic features in fundus eye examinations—bone spicule formation in the peripheral retina, blood vessel attenuation and pallor of the optic disc [4]. As there are no effective treatments for RP worldwide, recent research is focusing on novel therapeutic strategies, including gene augmentation therapy, cell transplants, and electronic prosthesis [5,6]. Moreover, future clinical trials will require additional novel outcome measures in order to assess responsibility for the treatment.

It is very likely that RP also affects other components of the visual pathway apart from the retina. Middle-aged (52.43 years) patients with RP exhibited abnormal brain network activity in various brain regions, as measured with functional magnetic resonance imaging (fMRI) [7]. Visual functioning, as measured using the voxel-wise degree centrality DC values of 16 patients with RP, was reduced in the right medial frontal gyrus, bilateral cuneus, bilateral precuneus, and bilateral superior frontal gyrus, and increased in the right cerebellum posterior lobe, left inferior temporal gyrus, and right fusiform gyrus. Moreover, voxel-based morphometry was applied to study whole-brain gray-matter volume changes in 27 RP patients with partially preserved vision in order to determine whether peripheral visual loss can lead to changes in gray-matter volume [8]. Significant reductions in gray-matter volume were found that were restricted to the occipital cortices of RP patients. It was significantly correlated with the extent of the peripheral visual field deficit in this cohort.

The lateral geniculate nucleus (LGN) has become a research target for many scientists due to its location, size, and complex functions in the visual pathway. The LGN is a part of the thalamus—a gray-matter structure located deep within the brain and serving as a final relay and modulation center for sensory information between the peripheral nervous system and the higher brain centers. Some data strongly suggest that abnormalities in the volume of thalamic nuclei indicate that a disease that affects the eye can cause changes not only in the white matter areas, including visual tracts, but also in the gray-matter structures [9,10]. However, the pathophysiological essentials of the central nervous system in RP are not well understood. Neurodegeneration beyond the visual pathway as atrophy of LGN has been shown in patients suffering from RP related to pathogenic variants in the RPGR gene [10], but further investigation, e.g., correlation with ophthalmological measures of visual function, is necessary.

A full understanding of LGN structure is only possible with ultra-high-resolution imaging due to its tiny size. However, routine structural magnetic resonance imaging (MRI) currently lacks the resolution and accuracy needed for exact LGN visualization and volume quantification. Overcoming these limitations, a 7 Tesla MRI examination allows for precise volumetric analysis of the LGN, despite its small size. To achieve these goals, we applied submillimeter high-field MRI for the evaluation of morphometric changes in the LGN in patients with advanced RP. The high spatial resolution at the 7T imaging level allows for accurate and possibly superior delineation of the LGN, based on high-resolution anatomical datasets, compared to standard imaging at 3T with the same acquisition time [9]. The 7T imaging specifically improves visualization of the LGN compared to 3T by utilizing the higher signal-to-noise ratio (SNR) to achieve higher spatial resolution and contrast, which is necessary to overcome the challenges posed by the small size, low contrast, and complex anatomical location of the LGN. 

The main aim of this study was to evaluate the volume of the LGN in patients with advanced late-stage RP disease using 7 Tesla MRI.

## 2. Materials and Methods

### 2.1. Subjects

This research was approved by the local medical ethics committee of the Medical University of Lublin (KE-0254/246/2020) and was carried out in compliance with national legislation and the Declaration of Helsinki. Patients were recruited in the out-patient clinic of the Department of General and Pediatric Ophthalmology of the Medical University of Lublin, Poland. The 7 Tesla scans of the brain were obtained at the Ecotech Complex in Lublin, Poland. Inclusion criteria were as follows: clinical diagnosis of advanced RP based on characteristic phenotype, visual acuity less than 0.1 according to Snellen charts, and age over 18 years old. Clinical features of RP are as follows: night blindness (nyctalopia); peripheral-visual-field constriction; and characteristic fundus findings: bone-spicule pigment deposits, retinal arteriole attenuation, and a waxy pale optic disc, as well as severely diminished or absent rod-driven responses (a-wave and b-wave) in electrophysiology examination. Disease duration was assessed and noted in months, based on the interview with the patient.

Seven females and twelve male participants were finally recruited for the study. The average age of the studied group was 52.43 years. Exclusion criteria were as follows: intraocular pathologies that would affect visual acuity or visual field, a history of ocular trauma, or surgery. Patients with any metallic implant, who were pregnant or breastfeeding, suffering from pathological changes within the cerebrovascular system as well as those suffering from claustrophobia, were excluded from the study. Radiological assessment had been performed by an experienced neuroradiologist (25 years of experience) and a neuroanatomy specialist (40 years of experience). An age-matched control group (21 participants) was recruited from the local community of medical workers after the clinical group was completed, in order to guarantee a demographic match of individuals from both samples. All of the participants were right-handed non-smokers with no history of chronic alcohol consumption. All participants signed informed consent.

### 2.2. MRI Acquisition

Magnetic resonance imaging (MRI) for this study was conducted at the ECOTECH Complex in Lublin, Poland, using a Discovery MR950 7 Tesla scanner (GE Healthcare, Chicago, IL, USA). The system operated with a maximum gradient strength of 50 mT/m and a slew rate of 200 T/m/s. Radio-frequency transmission was performed using a dual-channel birdcage coil driven in quadrature, whereas signal reception was accomplished with a 32-channel head array coil (Nova 2Tx/32Rx). The imaging protocol consisted of a non-contrast, three-dimensional sequence acquired at MRI 7T: 3D BRAVO T1-weighted. Detailed acquisition parameters are presented in Table 1.

The scan covered a field of view measuring 220 × 220 × 180 mm, with an acquisition matrix of 256 × 256 × 180. The resulting images were converted to a matrix of 512 × 512, achieving a voxel resolution of 0.43 × 0.43 × 1 mm. The imaging parameters included a TE of 2.6 ms, TR of 6.6 ms, and TI of 450 ms, with a flip angle of 12 degrees and a bandwidth of ±31.25 kHz. Parallel imaging was applied using an ARC acceleration factor of 2.

### 2.3. Image Analysis

**Figure 1:**
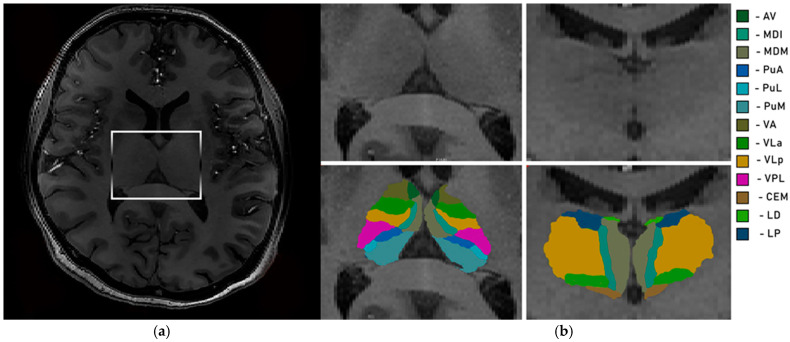
Segmentation of thalamus. Axial (**a**) and coronal (**b**) views of the thalamus. Av—anteroventral; MDI—mediodorsal lateral parvocellular; MDM—mediodorsal medial magnocellular; PuA—pulvinar anterior; PuL—pulvinar lateral; PuM—pulvinar medial; VA—ventral anterior; VLa—ventral lateral anterior; VLp—ventral lateral posterior; VPL—ventral posterolateral; CEM—central medial; LD—laterodorsal; and LP—lateral posterior.

**Figure 2:**
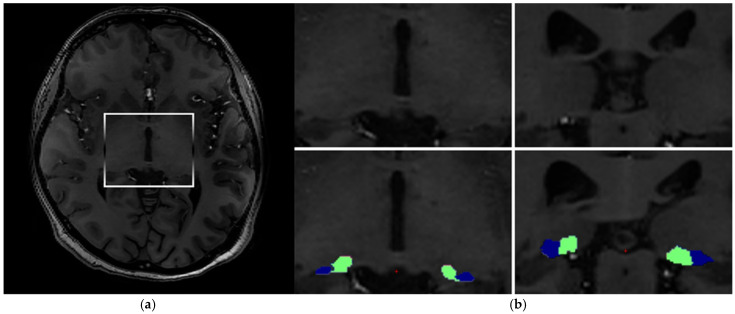
Segmentation of lateral geniculate nuclei LGN (blue) and medial geniculate nuclei MGN (green). Axial (**a**) and coronal (**b**) views of the nuclei.

Due to the pronounced field inhomogeneities inherent to 7T MRI, each structural scan of the brain underwent intensity bias correction using the unified segmentation algorithm implemented in SPM12 (http://www.fil.ion.ucl.ac.uk/spm, accessed on 20 December 2025; MATLAB R2018A, MathWorks, Natick, MA, USA). Brain segmentation was carried out with the “recon-all” function in the FreeSurfer software package (version 7.4.1, Massachusetts General Hospital, Harvard Medical School; http://surfer.nmr.mgh.harvard.edu/, accessed on 20 June 2023). To ensure stable processing, the original voxel size was resampled to 0.5 mm^3^. The number of surface inflation iterations was set to 100 and applied using the –cm flag within recon-all. The recon-all pipeline included signal intensity normalization, skull stripping to delineate skull structures within the normalized space, and automated segmentation. Following preprocessing, a radiologist performed a quality check. The experts were responsible solely for the qualitative visual inspection of the MRI scans to exclude unspecific brain pathologies and to verify the quality of the automated segmentation. All volumetric measurements were performed fully automatically by the software. Each image slice was visually examined for issues such as errors in skull stripping, segmentation, normalization, pial surface reconstruction, and topological inconsistencies, in accordance with FreeSurfer’s guidelines. Any datasets showing such issues were reprocessed with necessary corrections. In the subsequent analysis phase, segmentation of individual thalamic nuclei was performed. We extracted the absolute volumes of individual thalamic nuclei using a Bayesian segmentation technique that leverages a probabilistic atlas derived from histological data (Figure 1 and Figure 2) [11].

### 2.4. Statistical Analysis

To compare the study groups in terms of basic demographic variables, a two-tailed Student’s *t*-test was applied for quantitative measures, while the non-parametric χ^2^ test was employed for categorical characteristics. For the examination, between-group effects on volumetric data analysis of covariance (TIV) were reviewed, with age, total intercranial volume and sex included as covariates in group comparisons. For ANCOVA, the significance level was adjusted for multiple testing, and effects were considered statistically significant at *p* < 0.05. Partial eta squared (η*p*^2^) was reported as the measure of effect size. Subsequently, volumetric variables that significantly differentiated the groups were correlated with selected clinical (e.g., disease duration) and ophthalmologic measures, (e.g., BCVA) using Pearson’s r test, with correction for false discovery rate (FDR). Correlations were examined exclusively in the RP cohort. Prior to conducting the parametric tests, the assumption of data normality was assessed using the Shapiro–Wilk test, which indicated deviations from a normal distribution. Nevertheless, we proceeded with parametric analyses (i.e., independent samples *t*-test and ANCOVA). This approach is justified by the comparable sample sizes between our study groups (n = 19 for the RP group and n = 21 for the healthy control group) and the widely acknowledged robustness of the General Linear Model (including ANOVA and ANCOVA) to violations of the normality assumption under such conditions [12].

## 3. Results

### 3.1. Participants

Demographic and clinical data for the studied groups are presented in Table 2. Samples did not differ significantly in terms of age (RP = 48.48 and HC = 52.43) or sex (RP = 61% male and HC = 72% male). In the RP group, the duration of illness lasted for about 357 months (SD 114.87). Best-corrected visual acuity (BCVA) was significantly reduced in the RP group for both eyes compared to controls (*p* < 0.001).

### 3.2. Between-Group Comparisons

**Figure 3:**
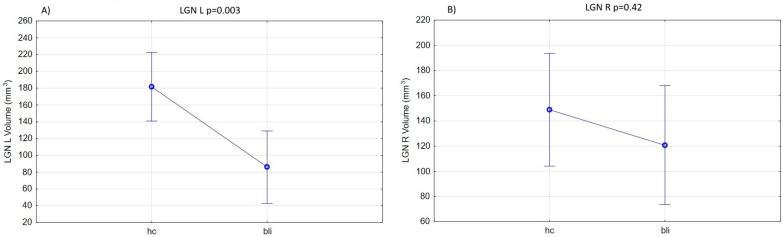
Between-group comparisons of the lateral geniculate nucleus (LGN): (**A**)—left and (**B**)—right volumes (mm^3^). The Figures show the mean, standard error, the range of 1.96 standard error and the level of statistical difference based on ANCOVA computations (abbreviations: hc—healthy controls group and bli—blind patients due to retinitis pigmentosa).

The RP and control groups did not differ significantly in terms of age or sex. Given the specificity of volumetric data, which require precise measurements of relatively small structures, group comparisons were performed while controlling for these two demographic characteristics, and the total intercranial volumes were used as covariates. The volumes of the left and right LGN were significantly smaller in the patients with RP, in comparison to those in the control sample: LGN left—F(1, 26) = 10.543, *p* = 0.003, η*p*^2^ = 0.23 and LGN right—F(1, 26) = 0.66, *p* = 0.42, η*p*^2^ = 0.018. After the ANCOVA analysis, only the left LGN was significantly different across the groups, with a large effect size of the disease on thalamic structural changes (the partial eta-squared values = 0.23). Figure 3A,B show the scope of the difference.

### 3.3. Volumetric—Clinical Correlations in RP Sample

**Figure 4:**
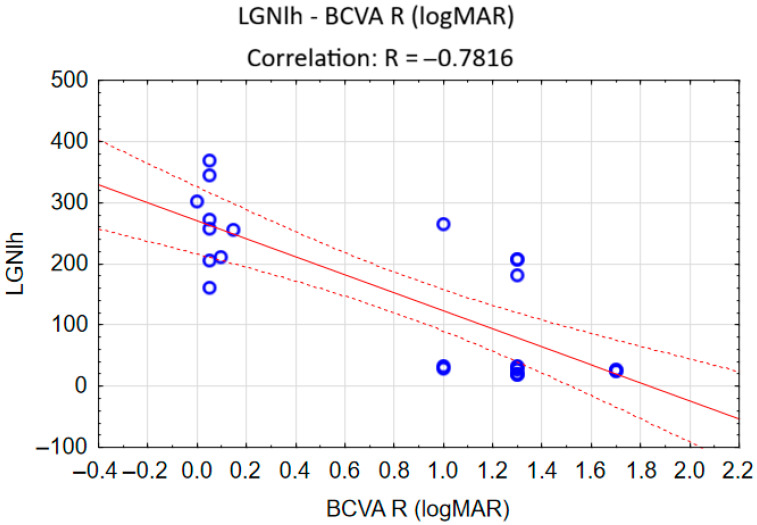
A correlation scatterplot showing associations between the volume of the left LGN and the right eye best-corrected visual acuity (BCVA R logMAR) in the RP group. Each blue circle represents the intersection of two numeric variables (LGN volume and logMAR visual acuity) for a single patient with retinitis pigmentosa. The red line is a trend line representing the negative correlation of relationship between two numerical variables (LGN volume and logMAR visual acuity). The red dotted line shows confidence intervals around the slope.

Among all the variables significantly separating the groups’ clinical characteristics, only the correlation between the volume of the left LGN and the right eye best-corrected visual acuity in the RP group was statistically significant: r = −0.7816 and *p* < 0.001. Figure 4 shows the correlations’ scatterplot.

## 4. Discussion

In this study, we applied submillimeter ultra-high-field MRI morphometry to the thalamic nuclei segmentation. It made it possible to perform precise delineation of the LGN areas in patients with advanced RP. Moreover, we have demonstrated that abnormalities within this part of the visual pathway were associated with selected features of the disease’s clinical picture, such as visual acuity in the right eye.

The most important finding of this study was the significantly reduced volume of the left LGN in patients with RP in comparison to those in the control group. Furthermore, we have also found that the volume of the left LGN was significantly correlated with right eye visual acuity in the RP patients. The contralateral association between the left LGN volume and the right eye visual acuity can be anatomically explained by the partial decussation of optic nerve fibers at the optic chiasm, where the majority of fibers from the nasal retina of the right eye cross over to the left hemisphere.

The LGN is a structure located in the metathalamus, and it acts as a transmitter of the visual pathway, providing an interconnection of the optic nerve as well as the optic tract and optic radiation. The results of this study showed that the volume of the LGN had significantly decreased in the advanced RP patient group compared with the control group. The LGN has also been found to be smaller in 20 patients with Usher syndrome, which is syndromic RP [13]. Moreover, additional structures of the brain that are engaged in visual processing have been measured in this study and found to be smaller in patients with Usher syndrome. Namely, the pericalcarine cortex, lingual gyrus, and cuneus, as well as those involved in hearing and language processing, such as the parsorbitalis and the rostral anterior cingulate cortex. Furthermore, patients with RP due to RPGR pathogenic variants have been assessed by the same group of researchers [10]. The result was that LGN and left lingual gyrus volumes were smaller and left isthmus cingulate and entorhinal cortices were higher in comparison to the control group. These data support the thesis of reorganization of the structures of the brain demonstrating a certain degree of plasticity in response to visual loss caused by RPGR-related RP. Fractional anisotropy (FA) measured by diffusion tensor MRI has also been used to assess the optic radiation in RP patients [14]. FA values of the bilateral anterior and posterior optic radiations were lower in patients with RP in comparison to controls.

Also, in another retinal dystrophy affecting the macula, Stargardt disease, the average LGN volume in both hemispheres was significantly smaller than in the control group [15]. Thus, a decrease in LGN size has been observed in both peripheral and central dystrophies of the retina, affecting both rods, cones and retinal pigment epithelium. It appears to be an unspecific sign of retinal degeneration.

Such findings were also described in optic nerve diseases, for example, normal tension glaucoma [16]. The LGN volume had significantly decreased in these patients compared to the controls. Similar results were also shown in Leber hereditary optic neuropathy (LHON) patients [17]. The potential benefits of the volumetry of LGN were noticed for patients with both normal tension [9] and open angle glaucoma [18]. In both research groups, the LGN volume was more significantly reduced than in controls. Kosior-Jarecka and colleagues also concluded that the LGN volume decreases during the course of glaucoma [18].

The LGN is a very important structure in the visual pathway, but it has not been investigated precisely enough because of its tiny size and the inability to measure it during standard MRI of the brain. It seems to be of major importance in retinal dystrophies that progress during life and lead to blindness. Vision relies on two types of photoreceptor cells, rods and cones, which are the first neurons in the visual pathway [19,20]. Photon absorption and signal transduction in the retina initiate visual processing. In phototransduction, photoreceptors convert photon information into electrical signals via the phototransduction cascade. These signals are transmitted through retinal neurons’ LGN and, finally, to the visual cortex [21]. Visual information from the retina reaches the LGN, which is located in the posteroventral thalamus, adjacent to the pulvinar and posterior to the inferior choroidal point. Its name derives from its position relative to the medial geniculate nucleus and its sharply bent laminae. The LGN comprises three main cell types: magnocellular (M), parvocellular (P), and koniocellular (K) [22]. It generates optic radiations (Meyer’s loop, central bundle, and Baum’s loop), projecting via the internal capsule to the primary visual cortex (V1). The LGN also modulates attention, supports binocular processing through monocular gain control, and performs temporal decorrelation. Some K cells exhibit orientation selectivity, akin to V1 neurons [22]. It has already been demonstrated that retinal, visual pathway, and visual cortical responses to light stimulation improve dramatically following retinal gene therapy in the canine model of *RPE65*-related Leber congenital amurosis, which is considered the most severe and early onset RP for which gene therapy treatment is already available [23]. Previous neuroimaging studies have primarily focused on functional reorganization following blindness in humans suffering from RP [24,25,26,27]. In adult patients with residual central vision, the occipital cortex exhibits an apparent reorganization of retinotopic maps [28].

While RP is traditionally viewed as a photoreceptor-specific disease, recent evidence confirms it is a neurodegenerative disease affecting the entire visual pathway, including retinal ganglion cells (RGCs) and the optic nerve, due to neural remodeling and trans-synaptic changes [29]. Damage to the retinal ganglion cells in the retina leads to degeneration of the visual cortex (anterograde degeneration) and vice versa (retrograde degeneration). The underlying mechanisms of the trans-synaptic degeneration are unknown; however, the form and extent of the degeneration can vary. Trans-synaptic anterograde degeneration will lead to changes in the LGN, optic radiation, and visual cortex, whereas trans-synaptic retrograde degeneration will affect the inner retina [27].

An important result of the present study is the correlation between the visual acuity in the right eye and the volume of the LGN in patients in the advanced RP group. This may be proof that LGN volume decreases as visual acuity declines. The above studies, along with those presented, may indicate that damage to the first neuron of the visual pathway results in changes in the volume of the LGN. From a clinical point of view, it therefore seems important to investigate, in future studies, whether outcomes in vision recovery after gene therapy or the use of retinal prostheses are related to changes in the volume of the LGN. A positive correlation would indicate neurodegeneration in the central nervous system. Moreover, to analyze the relationship between the volume loss of the LGN and changes in subsequent parts of the visual pathway, hybrid research combining submillimeter morphometry and the diffusion imaging should be conducted in patients with RP.

The limitation of this study was the relatively small group size (19 patients), which might at least partially reduce the statistical power of the main findings. However, similar studies also used small sample sizes due to the low prevalence of rare eye diseases [8,9,10,13]. Future research should expand the size for more accurate results. Some of the participants did not undergo genetic testing, which would have confirmed the diagnosis established based on clinical features. However, RP is very rare disease among the Polish population and variations in genotypes result in similar visual disturbances and clinical appearance, especially in end-stage disease. Another limitation is that images were obtained from 7 Tesla MRI used solely for research purposes, and the method of measuring LGN volume was automated.

## 5. Conclusions

The high precision of 7T MRI neuroimaging could reveal some significant associations between brain anomalies and selected features of the clinical picture of RP. Establishing such significant relationships could deepen current knowledge of the neuroanatomic basis of RP. The decrease in the volume of the LGN in advanced RP may reflect the trans-synaptic anterograde degeneration y in the visual pathway. Further studies are required for making a comparison of early-stage and late-stage RP in terms of LGN volume.

## Figures and Tables

**Table 1:** The 7T MRI acquisition parameters used in imaging of the brain for this research. Abbreviations: FOV (field of view), TE (echo time), TR (repetition time), TI (inversion time), and NEX (number of excitations).

	3D BRAVO T1-W
Scan duration	4 min 24 s
FOV [cm]	22 × 22
Slice thickness [mm]	1.0
TE [ms]	2.6
TR [ms]	6.6
TI [ms]	450
Matrix size	288 × 288
NEX	1
Flip Angle	12

**Table 2:** Demographic and clinical data of the study and control groups (M—mean; SD—standard derivation; χ^2^—chi-square distribution; BCVA—best-corrected visual acuity; and t-Student’s *t*-test).

	Retinitis Pigmentosa Group (n = 19) M (SD)	Control Group (n = 21) M (SD)	t Value or χ^2^	*p* Value
Age (years)	52.43 (7.42)	48.48 (14.41)	−0.54	0.59
Gender (% male)	61	72	−0.88	0.38
Duration of illness (months)	357 (114.87)			
Visual acuity left eye (Snellen) logmar	1.247 (0.367)	0.027 (0.026)	−9.094	*p* < 0.001
Visual acuity right eye (Snellen) logmar	1.293 (0.351)	0.051 (0.027)	−11.867	*p* < 0.001

## Data Availability

The original data presented in the study are openly available in https://repod.icm.edu.pl/dataset.xhtml?persistentId=doi:10.18150/SJAIBR, accessed on 4 March 2026.
